# Distinguishing highly-related outbreak-associated *Clostridium botulinum* type A(B) strains

**DOI:** 10.1186/1471-2180-14-192

**Published:** 2014-07-16

**Authors:** Brian H Raphael, Timothy B Shirey, Carolina Lúquez, Susan E Maslanka

**Affiliations:** 1 Enteric Diseases Laboratory Branch, Centers for Disease Control and Prevention, Atlanta, GA 30329, USA

**Keywords:** Botulism, Pulsed-field gel electrophoresis, Multi-loci variable number of tandem repeat analysis, Comparative genomic analysis, Single nucleotide polymorphism

## Abstract

**Background:**

In the United States, most *Clostridium botulinum* type A strains isolated during laboratory investigations of human botulism demonstrate the presence of an expressed type A botulinum neurotoxin (BoNT/A) gene and an unexpressed BoNT/B gene. These strains are designated type A(B). The most common pulsed-field gel electrophoresis (PFGE) pattern in the *C. botulinum* PulseNet database is composed of A(B) strains. The purpose of this study was to evaluate the ability of genome sequencing and multi-loci variable number of tandem repeat analysis (MLVA) to differentiate such strains.

**Results:**

The genome sequences of type A(B) strains evaluated in this study are closely related and cluster together compared to other available *C. botulinum* Group I genomes. *In silico* multilocus sequence typing (MLST) analysis (7-loci) was unable to differentiate any of the type A(B) strains isolated from seven different outbreak investigations evaluated in this study. A 15-locus MLVA scheme demonstrated an improved ability to differentiate these strains, however, repeat unit variation among the strains was restricted to only two loci. Reference-free single nucleotide polymorphism (SNP) analysis demonstrated the ability to differentiate strains from all of the outbreaks examined and a non-outbreak associated strain.

**Conclusions:**

This study confirms that type A(B) strains that share the same PFGE pattern also share closely-related genome sequences. The lack of a complete type A(B) strain representative genome sequence hinders the ability to assemble genomes by reference mapping and analysis of SNPs at pre-identified sites. However, compared to other methods evaluated in this study, a reference-free SNP analysis demonstrated optimal subtyping utility for type A(B) strains using *de novo* assembled genome sequences.

## Background

Botulism is a paralytic disease caused by the action of botulinum neurotoxins (BoNT) at neuromuscular junctions. BoNTs are produced by various botulinum toxin producing clostridia (BTPC) including *Clostridium botulinum* and some strains of *C. butyricum* and *C. baratii*[1]. Botulism can occur when food contaminated with BoNT is ingested or when organisms producing BoNT colonize the intestine (eg. infant botulism, adult intestinal colonization botulism) or when these organisms infect and produce toxin in wounds (eg. wound botulism due to traumatic injury or injection drug use) [1].

*C. botulinum* are classified into four Groups (I-IV) which are distinguished by biochemical properties and phylogenetic differences [2]. All strains of *C. botulinum* that produce BoNT/A belong to Group I. Nucleotide sequencing of the genes encoding BoNT/A reveals the presence of several toxin subtypes (eg. A1-A5) which form unique clades [3-5]. Some type A strains that produce BoNT/A also harbor an unexpressed gene encoding BoNT/B and are designated type A(B) [6].

Between 2010 and 2013, 47 *C. botulinum* type A strains were isolated during 36 laboratory investigations of botulism at the Centers for Disease Control and Prevention (CDC). Among these strains, 86% were determined to possess an unexpressed BoNT/B gene suggesting that type A(B) strains are common among US botulism cases (unpublished data). The *bont/A1* nucleotide sequence associated with type A(B) strains is highly conserved and contains only two nucleotide substitutions compared to the *bont/A1* sequence found in type A strains without an unexpressed *bont/B*[3]. A focused microarray featuring selected oligonucleotide probes based on the *C. botulinum* type A strain ATCC3502 genome sequence was unable to distinguish a group of unrelated type A(B) strains which suggested that these strains may share a high degree of genomic content [7]. Using a larger DNA microarray featuring probes targeting coding sequences in the ATCC3502 genome sequence, Carter et al. [4] demonstrated that several type A(B) strains isolated in North America shared a high degree of genomic content. Analysis of diverse panels of *C. botulinum* strains using multi-loci variable number of tandem repeat analysis (MLVA) and amplified fragment length polymorphism (AFLP) demonstrate that type A(B) strains cluster separately from strains encoding other *bont/A* subtypes and from strains expressing BoNT/A1 but lacking an unexpressed *bont/B*[3,8]. More recently, a standardized *C. botulinum* pulsed-field gel electrophoresis (PFGE) method has been established ( http://www.cdc.gov/pulsenet/PDF/c-botulinum-protocol-508c.pdf). A national subtyping database (PulseNet) of PFGE patterns using this method is maintained at CDC and individual patterns are given unique identifiers. The most frequent *SmaI* PFGE pattern (30%) observed among 256 *C. botulinum* types A, B, E, and F strains examined has been designated DRPS16.0001. Additional characterization of these strains revealed that they are type A(B) and all share the same PFGE *XhoI* pattern (DRPX11.0001).

During laboratory investigations of botulism, specimens from different sources may be examined. For instance, in a foodborne botulism outbreak, clinical samples (such as serum and stool) from one or more individuals as well as suspected food sources may be submitted for testing. Toxin detection either in clinical samples or food ingested by individuals with clinical symptoms of botulism is sufficient for laboratory confirmation [1]. Additionally, BTPC may also be isolated from both stool and food. Isolation of BTPC from stool of individuals with botulism provides ancillary evidence of toxin serotype when toxin is also detected in clinical specimens. In some cases, isolation of BTPC in stool of individuals with symptoms of botulism provides the only evidence for laboratory confirmation in clinical specimens. Similarly, isolation of BTPC from foods provides supportive results for the identification of toxin in a contaminated food. However, isolation of BTPC only in a food source is insufficient for laboratory confirmation due to the ubiquity of such organisms in the environment. In some investigations, left over food actually consumed by patients is not available but equivalent lots may be recovered from the home or manufacturer. Therefore, subtyping methods that permit comparison of BTPC isolates from different sources have an important role in supporting epidemiological links among samples examined during a laboratory investigation of botulism especially in cases where toxin cannot be detected directly in the sample submitted for testing.

In this study, type A(B) strains isolated from different sources among separate foodborne botulism outbreaks occurring in the US were selected for study because they shared indistinguishable *SmaI* and *XhoI* PFGE patterns (Table 1). An additional type A(B) strain (CDC42961) that was unrelated to any of the botulism outbreaks was selected as an outlier. We examined the ability of high resolution subtyping methods including MLVA and genome sequence analysis (e.g. fragmented genome alignment, *in slico* multilocus sequence typing (MLST) analysis, reference-free SNP analysis) to distinguish these type A(B) strains isolated from separate botulism outbreaks yet sharing a common PFGE pattern. This work confirms that these type A(B) strains are highly-related and evaluates the utility of methods for clustering *C. botulinum* type A(B) strains isolated from separate sources during an outbreak investigation.

**Table 1:** Characteristics of strains used in this study

**Outbreak**	**Reference**^**a**^	**Strain**	**Source**	**Origin**	**Year isolated**	***SmaI *** **PFGE pattern**	***XhoI *** **PFGE pattern**
1	This study	CDC28012	Stool	Utah	1973	DRPS16.0001	DRPX11.0001
		CDC28023	Chili	Utah	1973	DRPS16.0001	DRPX11.0001
2	This study	CDC33700	Stool	Florida	1988	DRPS16.0001	DRPX11.0001
		CDC33702	Seafood pasta	Florida	1988	DRPS16.0001	DRPX11.0001
3	This study	CDC37457	Stool^b^	Alaska	1982	DRPS16.0001	DRPX11.0001
		CDC37461	Stool^b^	Alaska	1982	DRPS16.0001	DRPX11.0001
4	[18]	CDC48719	Stool	Georgia	1993	DRPS16.0001	DRPX11.0001
		CDC48761	Swab from cutting board	Georgia	1993	DRPS16.0001	DRPX11.0001
5	[19,20]	CDC52271	Chili sauce	Indiana	2007	DRPS16.0001	DRPX11.0001
		CDC52298	Stool	Indiana	2007	DRPS16.0001	DRPX11.0001
6	This study	CDC66088	Stool	New York	2011	DRPS16.0001	DRPX11.0001
		CDC66089	Cooked barley, sausage, cheese	New York	2011	DRPS16.0001	DRPX11.0001
7	[21]	CDC67187	Rectal Swab	Arizona	2012	DRPS16.0001	DRPX11.0001
		CDC67190	Pruno	Arizona	2012	DRPS16.0001	DRPX11.0001
NA^c^	This study	CDC42961	Unknown^d^	Ecuador	1997	DRPS16.0001	DRPX11.0001

^a^Where available, references shown describe a specific outbreak or investigation.

^b^Stool samples isolated from different individuals with foodborne botulism associated with "stink eggs".

^c^NA = not applicable; strain CDC42961 is not epidemiologically associated with any other strain examined in this study.

^d^Strain received at CDC as a culture.

## Methods

### Bacterial strains used in this study

*C. botulinum* strains used in this study are indicated in Table 1. Strains were grown in Trypticase Peptone Yeast Extract (TPGY; Remel, Lenexa, KS) at 35°C under anaerobic conditions. Two representative strains from different sources were selected from seven separate previous laboratory investigations. An additional strain (CDC42961) unrelated to any of these investigations was also examined.

For additional studies, some isolates associated with outbreak #5 are further identified as "picks" based on their original isolation from enrichment cultures of a single sample. The pick designation following the strain number refers to the enrichment culture from which a single colony isolate was derived as follows: H = heated (80°C, 15 minutes) Cooked Meat Glucose Starch (CMGS; Remel, Lenexa, KS), T = TPGY containing 0.09% trypsin, PL = untreated (plain) CMGS. Separate single colonies from each enrichment culture type are indicated by a digit following the pick designation. All strains were coded prior to the study and managed in compliance with a human subjects exemption protocol (#4991.0) approved by the CDC Human Research Protection Office.

### MLVA

MLVA was performed using the 15-loci scheme described by Fillo et al. [9]. Fragment analysis was performed as a separate reaction for each allele using a CEQ8000 Genetic Analysis System and the GenomeLab™ DNA Size Standard Kit – 600 (Beckman Coulter, Brea, CA). The largest peaks were identified in the fragment analysis and repeat units (RU) are expressed as the lowest number of intact units.

### Genome sequencing

Genomic DNA was extracted from overnight TPGY cultures as described previously [10] and further purified using the DNA Clean & Concentrator-5 kit (Zymo Research, Irvine, CA). Fragment libraries were constructed using 100 ng of genomic DNA with the Ion Xpress™ Fragment Library kit and size-selection was performed using the E-Gel® Agarose Gel Electrophoresis System (Life Technologies, Grand Island, NY). Library concentrations were determined using the Qubit® dsDNA HS Assay (Life Technologies, Grand Island, NY) and diluted to a final concentration of 18 pM. Templates were generated using the Ion OneTouch™ 200 Template Kit v2 and sequencing was performed using the Ion Torrent™ Personal Genome Machine (PGM™) with 314 v2 chips. Barcoded fragment libraries of various "picks" of strains CDC 52271 and CDC 52298 were generated with the Ion Torrent-compatible Bioo Scientific (Austin, TX) NEXflex™ DNA barcodes and sequenced with a 316 v2 chip.

### Bioinformatic analysis

Sequence reads were *de novo* assembled using MIRA v3.9.9 (performed with the Assembler plug-in on the Torrent Suite 4.0.2 software). Assembled genome metrics and GenBank accession numbers are shown in Table 2. Draft genomes were compared using Gegenees software which aligns fragments of each sequence using BLAST and generates a phylogenomic distance based on the average similarity score of fragments in pairwise alignments [11]. *In silico* MLST was performed using the MLST 1.7 tool available at Center for Genomic Epidemiology website ( http://cge.cbs.dtu.dk/services/MLST/) [12]. Single nucleotide polymorphism (SNP) discovery was performed on concatenated sequence data using the SNP detection program kSNP v1 [13] with a k-mer size of 21. Maximum parsimony trees were constructed in MEGA v5.2 [14] using core SNP data matrices, which include only SNPs detected at loci that were present in all genomes.

**Table 2:** Draft genome sequence properties

**Strain**	**# reads assembled**	**Genome size (Mb)**	**# contigs**	**N50 (kb)**	**Coverage (X)**	**GenBank accession number**
CDC28012	547,889	3.97	400	19.1	29	JFGN01000000
CDC28023	522,829	3.97	341	22.3	27	JFGM01000000
CDC33700	556,551	3.68	869	6.4	30	JFGL01000000
CDC33702	638,235	3.70	880	6.2	37	JFGK01000000
CDC37457	594,516	3.90	456	14.5	34	JFGJ01000000
CDC37461	502,932	3.90	389	18.4	29	JFGI01000000
CDC48719	625,316	3.98	310	26.1	29	JFGH01000000
CDC48761	646,981	3.96	418	17.0	36	JFGG01000000
CDC52271	519,456	3.89	399	16.9	28	JFGF01000000
CDC52298	502,934	3.87	524	12.0	28	JFGE01000000
CDC66088	801,959	3.83	1,086	5.6	42	JFGD01000000
CDC66089	458,975	3.44	1,093	4.1	26	JFGC01000000
CDC67187	462,117	3.56	1,079	4.4	27	JFGB01000000
CDC67190	594,808	3.73	1,046	5.0	35	JFGA01000000
CDC42961	681,808	3.89	684	9.4	35	JFFZ01000000

## Results and discussion

### Resolution of *C. botulinum* type A(B) strains using MLVA

Only 2 of the 15 loci (i.e. loci 1 and 4 reported by Filio et al. [9]) examined using MLVA displayed variation in RU among the strains examined in this study (Table 3). Outbreak-related strains shared identical MLVA profiles with the exception of strains composing outbreak #3 and outbreak #5 which had a divergent number of repeats at various loci. In addition, the strains composing some outbreaks could not be resolved from those isolated from separate outbreaks. More specifically, the strains associated with outbreaks #6 and #7 shared identical MLVA profiles. Moreover, strains composing outbreak #2 and the outlying strain CDC42961 shared the same MLVA profile. Taken together, these results demonstrate that MLVA only partially differentiates closely related type A(B) strains and not all strains associated with separate outbreaks could be resolved.

**Table 3:** MLVA results

	**Number of repeat units (RU)**														
Locus	1	2	3	4	5	6	7	8	9	10	11	12	13	14	15
CDC28012	9	–^a^	5	8	12	2	4	6	2	13	3	1	23	6	3
CDC28023	9	–	5	8	12	2	4	6	2	13	3	1	23	6	3
CDC33700	8	–	5	7	12	2	4	6	2	13	3	1	23	6	3
CDC33702	8	–	5	7	12	2	4	6	2	13	3	1	23	6	3
CDC37457	7	–	5	8	12	2	4	6	2	13	3	1	23	6	3
CDC37461	8	–	5	7	12	2	4	6	2	13	3	1	23	6	3
CDC48719	8	–	5	9	12	2	4	6	2	13	3	1	23	6	3
CDC48761	8	–	5	9	12	2	4	6	2	13	3	1	23	6	3
CDC52271	7	–	5	7	12	2	4	6	2	13	3	1	23	6	3
CDC52298	11	–	5	7	12	2	4	6	2	13	3	1	23	6	3
CDC66088	8	–	5	8	12	2	4	6	2	13	3	1	23	6	3
CDC66089	8	–	5	8	12	2	4	6	2	13	3	1	23	6	3
CDC67187	8	–	5	8	12	2	4	6	2	13	3	1	23	6	3
CDC67190	8	–	5	8	12	2	4	6	2	13	3	1	23	6	3
CDC42961	8	–	5	7	12	2	4	6	2	13	3	1	23	6	3

^a^Deletions are indicated by a "–"symbol

### Genome sequencing reveals that *C. botulinum* type A(B) strains are highly-related

**Figure 1:**
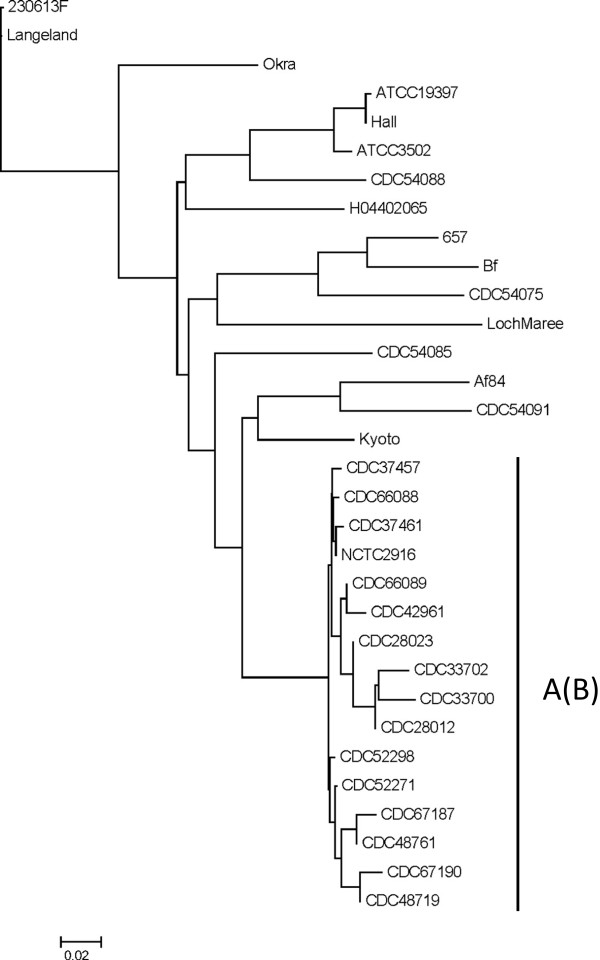
**Phylogenomic analysis of *C. botulinum *Group I strains.** *C. botulinum* Group I genome sequences were compared using a fragmented alignment and a neighbor joining tree of phylogenomic distances is shown. The cluster of type A(B) strains is indicated. The accession numbers of sequences obtained from the NCBI genomes database are as follows: GenBank:NC_017297 (230613 F), GenBank:NC_009699 (Langeland), GenBank:NC_010516 (Okra), GenBank:NC_012658 (657), GenBank:NZ_ABDP00000000 (Bf), GenBank:NZ_AOSX00000000 (Af84), GenBank:NC_012563 (Kyoto), GenBank:NZ_ABDO00000000 (NCTC2916), GenBank:NC_009697 (ATCC19397), GenBank:NC_009698 (Hall), GenBank:NC_009495 (ATCC3502), GenBank:NC_017299 (H04402065), GenBank:NC_010520 (LochMaree), GenBank:AZQW01000000 (CDC54075); GenBank:AZRQ01000000 (CDC54085); GenBank:AZRR01000000 (CDC54091); and GenBank:AZRS01000000 (CDC54088).

Draft genome sequences from the 15 type A(B) strains were compared along with several reference genomes selected from the National Center for Biotechnology Information (NCBI) database using a fragmented alignment (Figure 1). This comparison showed that all of the type A(B) genomes (including a separately sequenced type A(B) strain NCTC2916) were closely related and formed a unique clade. Within this clade, only one pair of outbreak-associated strains (CDC33700 and CDC33702) branched from a shared node indicating that such analysis is unsuitable for clustering outbreak-related strains that share a high degree of genomic similarity.

As shown in Table 4, *in silico* MLST analysis demonstrated that all 15 draft genome sequences matched identically (or nearly identically) with the alleles forming ST-4 in the MLST scheme reported by Jacobson et al. [15]. In some cases, nucleotide mismatches with the best-matched allele occurred (usually in homopolymer regions) or the draft sequence did not cover the entire best-matched allele (for instance when the allele sequence was truncated by the end of a contiguous sequence). Several type A(B) strains are associated with ST-4 including the reference genome sequence of strain NCTC2916 (Table 4) and 13 out of 24 type A(B) strains examined by Jacobson et al. [15]. In that study, ST-4 was composed exclusively of type A(B) strains.

**Table 4:** ***In silico *** **MLST best match results**

	**% Identity/% Coverage of allele**						
**Locus**^**a**^	***aceK***	***aroE***	***hsp***	***mdh***	***oppB***	***recA***	***rpoB***
**Allele**	**6**	**9**	**8**	**10**	**9**	**7**	**8**
CDC28012	100/100	100/100	100/100	100/100	100/100	100/100	100/100
CDC28023	100/100	100/100	100/100	100/100	100/100	100/100	100/100
CDC33700	100/100	100/100	100/100	100/100	100/100	100/100	100/100
CDC33702	100/100	100/100	100/100	100/100	100/100	100/100	100/100
CDC37457	100/100	100/100	100/100	100/100	100/100	100/100	100/100
CDC37461	100/100	100/100	100/90.4	100/100	100/100	100/100	100/100
CDC42961	100/100	100/100	100/100	100/100	100/100	100/100	100/100
CDC48719	100/100	99.8/100	100/100	100/100	100/100	100/100	100/100
CDC48761	100/100	100/100	100/100	100/100	100/100	100/100	100/100
CDC52271	100/100	100/100	100/100	100/100	100/100	100/100	100/100
CDC52298	100/100	100/100	100/100	100/100	100/100	100/100	100/100
CDC66088	100/100	100/100	100/100	100/96.0	100/100	100/100	100/100
CDC66089	100/100	100/100	100/100	100/100	99.6/100	100/100	100/100
CDC67187	100/100	99.8/100	100/93.4	100/100	100/100	100/100	100/100
CDC67190	100/100	100/100	100/100	100/100	100/100	100/100	100/100
NCTC2916	100/100	100/100	100/100	100/100	100/100	100/100	100/100

^a^Loci refer to those genes used in the MLST scheme developed by Jacobson et al. [15].

### Resolution of *C. botulinum* type A(B) strains using reference-free SNP analysis

**Figure 2:**
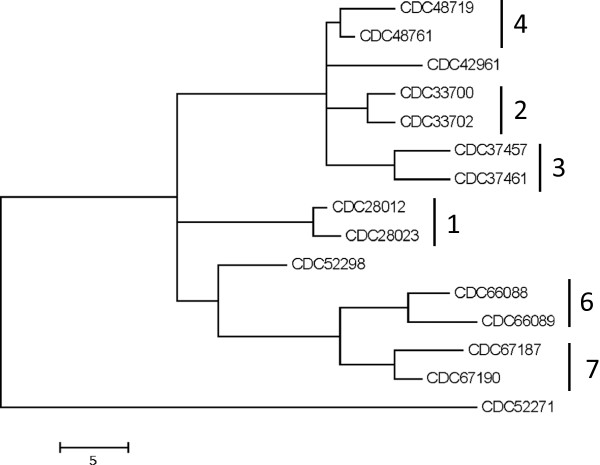
**SNP analysis of *C. botulinum *type A(B) draft genomes sequences.** Core SNPs were identified using kSNP and a maximum parsimony tree of the core SNP matrix is shown. Outbreak-related strains are indicated by outbreak number as shown in Table 1.

Previous work has demonstrated the utility of reference-free SNP analysis for the differentiation of *C. botulinum* Group I strains including those with identical BoNT subtypes [16]. This work was performed using the software program, kSNP, which can be used to identify SNPs among a set of draft and/or complete microbial genomes without the need for comparison to a reference genome [13,17]. Analysis of the 15 draft genomes generated in this study using kSNP revealed the presence of 145 core SNPs (i.e. SNPs detected at loci present in all genomes). Phylogenetic analysis of these SNPs demonstrated that strains associated with each outbreak generally clustered together (Figure 2) and differed by only 3–8 SNPs. However, the strains associated with outbreak #5 (CDC52271 and CDC52298) were more divergent and differed by 56 SNPs. Notably, strain CDC42961, which is not associated with any of the seven outbreaks examined, did not cluster with any other strain suggesting that this approach may successfully differentiate unrelated A(B) strains.

### Resolution of multiple isolates associated with outbreak #5

**Figure 3:**
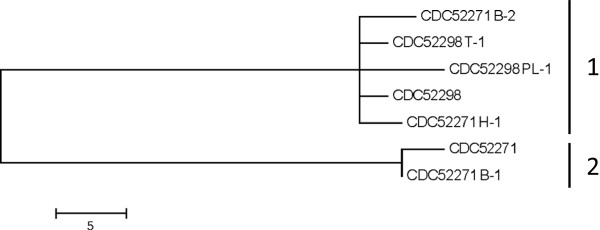
**SNP analysis of strains isolated from outbreak #5.** Core SNPs were identified using kSNP and a maximum parsimony tree of the core SNP matrix is shown. The sequences cluster into two groups as indicated in the figure.

In order to better assess the genetic diversity of the strains isolated from outbreak #5, several "picks" derived from single colonies originally isolated from different enrichment cultures of each sample (i.e. stool and food samples) were examined. Genome sequencing of these isolates revealed the presence of two groups (Figure 3). Group 1 contained the originally examined genome sequence of strain CDC52298 and two picks (CDC52298 PL-1 and CDC52298 T-1) from the same source (stool). Also included in Group 1 were picks (CDC52271 B-2, CDC52271 H-1) associated with the implicated food (chili sauce). Group 2 only contained the original strain CDC52271 genome sequence and one pick (CDC52271 B-1) associated with the same source. The number of SNPs differentiating strains associated with Groups 1 and 2 ranged from 56–63. The sequences of strains within Group 1 differed by only 4–10 SNPs and those composing Group 2 differed by only 3 SNPs. Notably, the MLVA profiles of CDC52298 PL-1, CDC52298 T-1, CDC52271 H-1, and CDC52271 B-2 were identical and also different than the profile of 52271 B-1 (data not shown), thus supporting the clustering of these isolates by SNP analysis. These data indicate that the contaminated food product contained at least two separate populations of type A(B) strains and that the strains isolated from stool of an individual who consumed the food were associated with only one these populations. These populations differ by multiple mutational events and are unlikely to be the result of recent divergence. However, more detailed comparative genomic analysis of these strains is hindered by the lack of a complete reference genome sequence.

## Conclusions

The *C. botulinum* type A(B) strains examined in this study which shared indistinguishable PFGE profiles also shared a high degree of genomic sequence similarity. While the type A(B) strains clustered together in a comparison of *C. botulinum* Group I genome sequences using a fragmented alignment, this method did not resolve strains associated with separate outbreaks. The lack of adequate resolution using this method may have been influenced by differences in the overall level of genome coverage among the *de novo* assembled genomes, which share little sequence variation. Strains isolated from separate foodborne botulism outbreaks also could not be distinguished by *in silico* MLST and only partially by MLVA.

These results suggested that genetic differences among this group of highly-related type A(B) strains may be restricted to a limited number of SNPs. A complete *C. botulinum* type A(B) genome sequence is not currently available thereby preventing the ability to perform reference alignments of sequencing reads and assessment of variation at specific SNP loci. However, reference-free SNP analysis of *de novo* aligned draft sequences effectively resolved strains isolated from 7 separate outbreaks and an outlying unassociated type A(B) strain. It is important to note that strains isolated from the same outbreak shared a low number of SNPs. It is unclear if these SNPs are associated with actual genetic variation or are artifacts of sequencing errors. Future work identifying high quality SNPs based on comparison with one or more reference genome sequences of such strains is needed.

In addition, this study demonstrates that a contaminated food product may contain multiple strains of *C. botulinum* some of which could be genetically distinguishable from clinical sample isolates. This finding suggests that subtyping methods may need to be applied to several isolates from the same source to ensure adequate sampling of multiple strain populations that may be present.

## Competing interests

The authors declare that they have no competing interests.

## Authors’ contributions

BHR and CL designed the study. BHR and TBS performed experiments and CL and SEM contributed to data analysis. BHR and TBS drafted the manuscript. All authors reviewed and approved the final manuscript.
